# Supplementary material for: Pulse Oximetry as an Aid to Rule Out Pneumonia among Patients with a Lower Respiratory Tract Infection in Primary Care
Source: Antibiotics (Basel). 2023 Mar 2;12(3):496. doi: 10.3390/antibiotics12030496 (PMC10044291; doi:10.3390/antibiotics12030496)
Supplement: Supplementary file 1 [file antibiotics-12-00496-s001.zip › antibiotics-2227641-supplementary.pdf]

Supplementary Table S1. Characteristics of the patients included in this ancillary study compared to those who were not included (because of the absence of recorded chest X-ray or vital signs) from the original cluster randomized trial.

	**All** N=469	**Not included** N=362 (77%)	**Included** N=107 (23%)	**p**
**Demographics and comorbidities**				
Female	278 (59)	213 (59)	65 (61)	0.810
Age ≥ 65 years	126 (27)	92 (25)	34 (32)	0.238
Active smoker	101 (22)	77 (21)	24 (22)	0.913
Any comorbidity	116 (26)	88 (25)	28 (27)	0.864
Chronic obstructive pulmonary disease	30 (6.4)	22 (6.1)	8 (7.5)	0.654
Asthma	70 (15)	51 (14)	19 (18)	0.448
Other comorbidity*	29 (6.4)	24 (6.8)	5 (4.8)	0.647
**Symptoms and signs**				
Sputum production	322 (69)	248 (69)	74 (70)	0.981
History of fever	317 (68)	243 (67)	74 (69)	0.782
History of dyspnoea	302 (65)	229 (64)	73 (69)	0.397
History of chest pain	197 (43)	151 (42)	46 (43)	1.000
Heart rate > 100/minute	66 (14)	49 (14)	17 (16)	0.663
Temperature ≥ 37.8°C	75 (16)	52 (14)	23 (22)	0.111
Oxygen saturation < 95%	93 (23)	64 (22)	29 (27)	0.300
Respiratory rate ≥ 24/minute	77 (17)	52 (15)	25 (23)	0.043
Hypotension**	24 (5.2)	16 (4.5)	8 (7.5)	0.219
CRB-65 ≥ 1 point	137 (30)	98 (28)	39 (36)	0.095
Abnormal lung auscultation	220 (47)	145 (41)	75 (70)	<0.001

Values are n (%).

*Other comorbidity: heart failure (4/469, 0.85%), diabetes (21/469, 4.5%), active malignancy (5/469, 1.1%), chronic kidney disease (0/469, 0.0%) or human immunodeficiency virus infection (0/469, 0.0%).

**Hypotension: systolic blood pressure ≤ 90 mmHg or diastolic blood pressure ≤ 60 mmHg
